# Extramammary Paget Disease Secondary to Axillary Apocrine Carcinoma: Highlighting the Diagnostic Utility of a Limited Immunohistochemical Panel in a Resource-Limited Setting

**DOI:** 10.7759/cureus.99185

**Published:** 2025-12-14

**Authors:** Ricardo Manuel Dávila

**Affiliations:** 1 Dermatology, Hospital Santo Tomás, Panama City, PAN

**Keywords:** adenocarcinoma, axillary empd, bowen disease differential diagnosis, chronic eczematous lesions, extramammary paget’s disease, intraepidermal pagetoid neoplasms, melanoma differential diagnosis, paget cells, rare axillary lesions, rare dermatologic malignancies

## Abstract

Extramammary Paget disease (EMPD) is a rare adenocarcinoma arising in apocrine gland-rich skin, most commonly affecting the genital and perianal regions, and may present as either a primary intraepidermal neoplasm or a secondary, epidermotropic extension of an underlying malignancy.

We report the case of a 54-year-old Panamanian man who presented with a two-month history of a right axillary plaque, characterized by a beefy-red, moist surface with moderate scaling and scattered erosions, accompanied by intermittent burning pain and pruritus. Dermoscopy revealed milky-red areas interspersed with cloud-like, structureless areas, prompting a differential diagnosis that included Bowen disease, malignant melanoma, and EMPD. Histopathology demonstrated intraepidermal nests of large, pale cells with vesicular, pleomorphic nuclei; these cells were positive for epithelial membrane antigen, cytokeratin 7, and cytokeratin 8/18, and negative for S-100 and Melan-A. Wide local excision with lymph node dissection confirmed EMPD secondary to an underlying apocrine carcinoma. Axillary EMPD is uncommon and may be clinically subtle, leading to delayed diagnosis, particularly in resource-limited settings where access to immunohistochemistry (IHC) is restricted. Chronic erythematous or pruritic plaques on apocrine-rich skin should prompt biopsy, as histology, coupled with IHC, is essential for accurate classification and management. To our knowledge, this represents the first documented case of axillary EMPD reported in Panama.

## Introduction

Extramammary Paget disease (EMPD) is an adenocarcinoma of apocrine gland-rich skin [1]. Mammary Paget disease (MPD), described first, is associated with breast cancer. The “extramammary” form shares clinical and histologic features but is considered a separate entity. EMPD can be primary or secondary. Primary EMPD is an intraepidermal neoplasm of glandular origin, whereas secondary EMPD is an epidermotropic metastasis of an underlying adenocarcinoma [2]. Associated internal malignancies include bladder, gastrointestinal, and prostate cancer, influencing prognosis [3]. Adnexal neoplasms have also been implicated [4-7]. The most common locations of EMPD are the genital and perianal regions [1]. Isolated axillary EMPD is a relatively rare entity that mostly affects patients of Asian ancestry [3]. To our knowledge, we present the first documented case of axillary EMPD in a Panamanian male. 

## Case presentation

**Figure 1:**
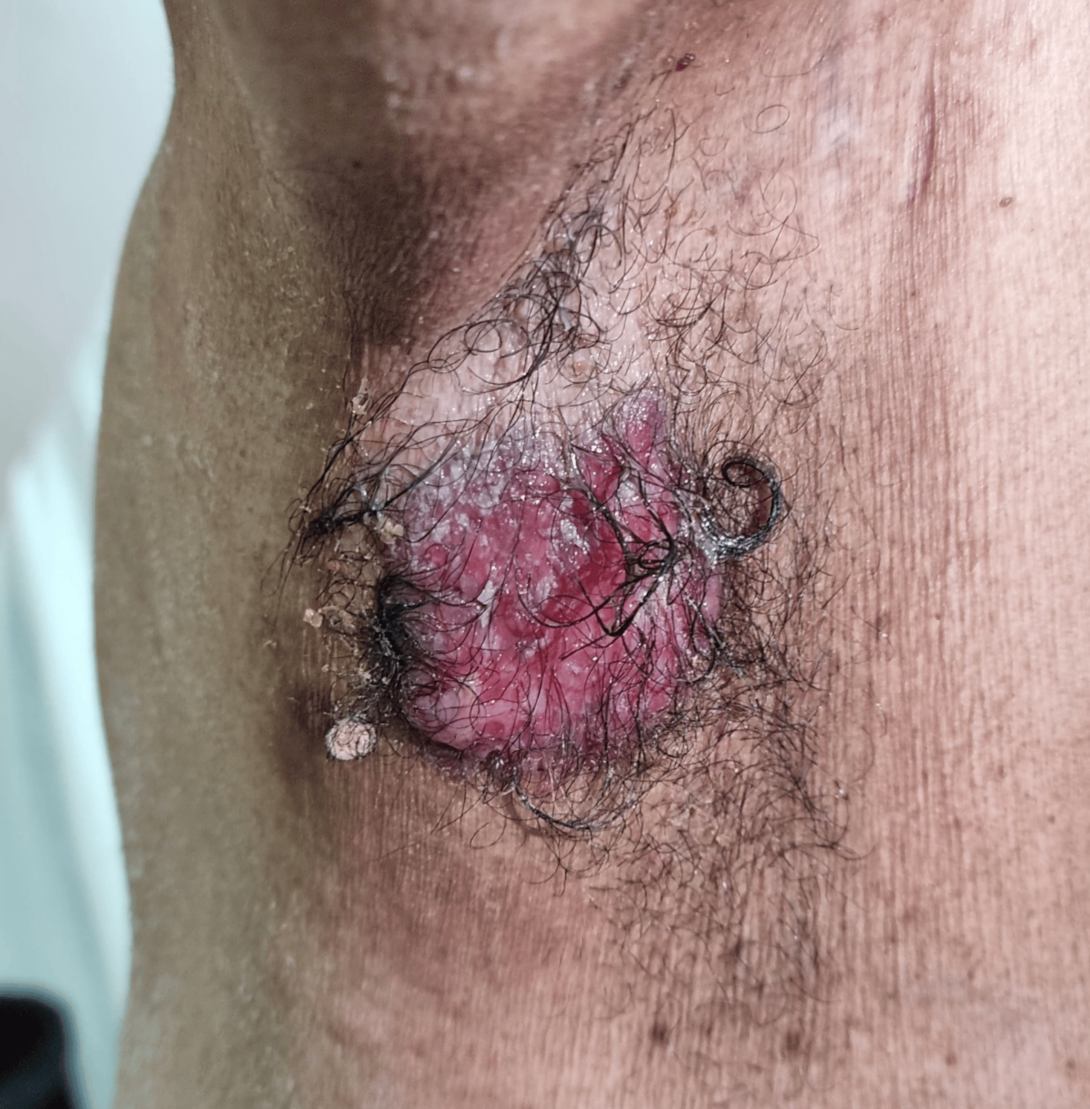
Right axillary plaque

**Figure 2:**
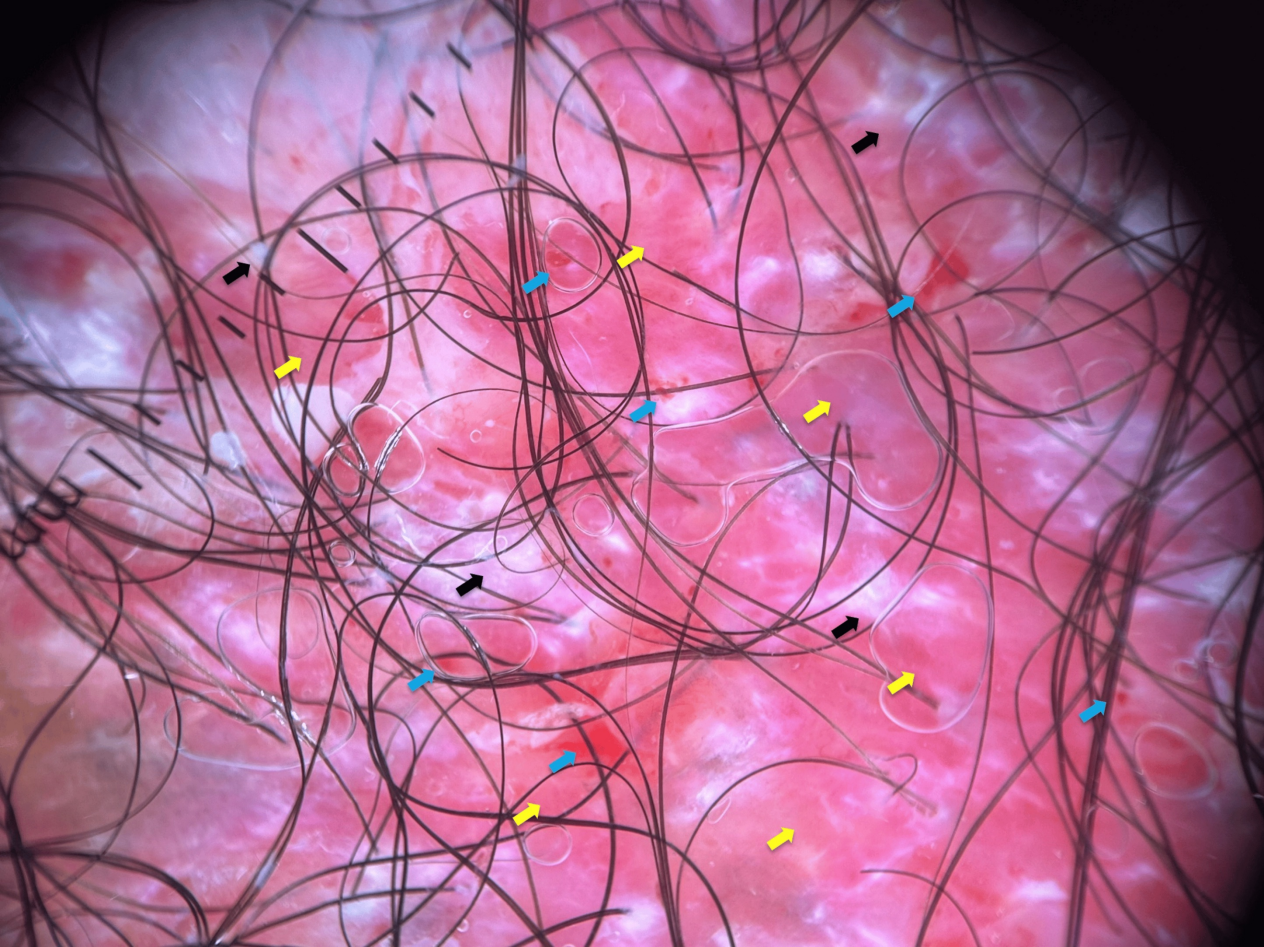
Polarized dermoscopy White, cloud-like, structureless areas (black arrows); milky-red areas (yellow arrows); erosions (blue arrows).

A 54-year-old Panamanian male, who was admitted due to a myocardial infarction, was seen by our team. His past medical and family history were unremarkable. He complained of a right axillary lesion, which he had noted two months earlier. Upon physical examination, an erythematous plaque measuring 7.0 × 5.5 cm was observed on the right axillary vault. The lesion had a moist, beefy-red surface, with moderate scaling and a few scattered erosions. Induration was evident (Figure 1). Upon further questioning, he reported occasional burning pain and pruritus. The patient had not experienced fever, weight loss, changes in bowel habits, rectal bleeding, dysuria, or hematuria. Examination of the breasts, the contralateral axilla, and the skin of the pubic, inguinal, genital, perineal, and perianal regions was within normal limits. Abdominal examination was noncontributory, with no visceromegaly or masses. Lymph nodes were of normal size. The rest of the examination was unremarkable. Milky-red areas, interspersed with white, structureless areas, were present upon dermoscopic examination (Figure 2).

Our differential diagnoses were Bowen disease, malignant melanoma, and EMPD.

**Figure 3:**
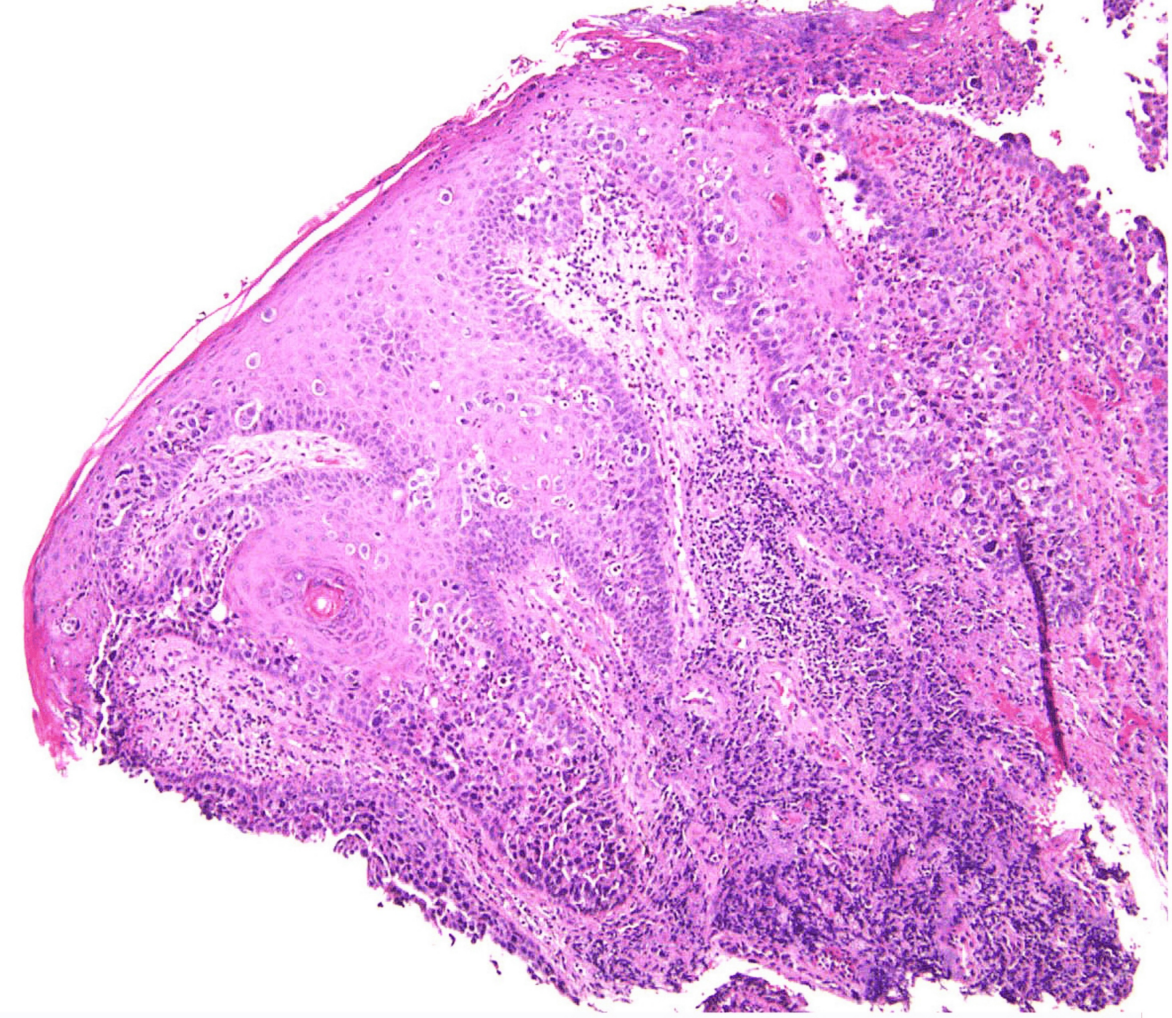
Hematoxylin & eosin stain

**Figure 4:**
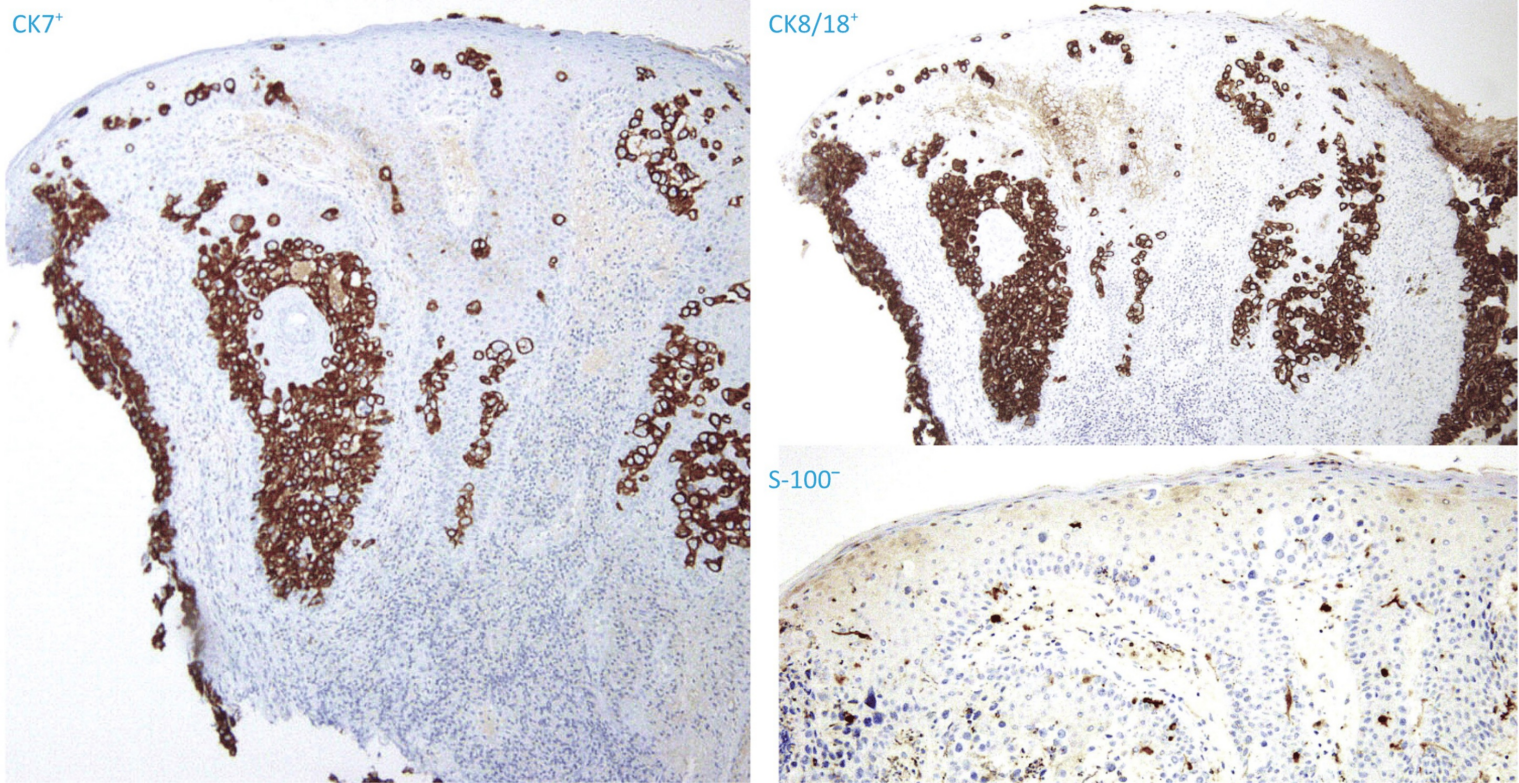
Immunohistochemistry Left: CK7 staining was positive; Top right corner: positive CK8/18; Bottom right corner: negative S-100. CK7: cytokeratin 7; CK8/18: cytokeratin 8/18

Two skin samples were obtained by punch biopsy and examined with H&E. Both specimens exhibited an epidermis with nests of cells containing large, pale cytoplasm and large, vesicular, pleomorphic nuclei; some were hyperchromatic (Figure 3). These cells were positive for epithelial membrane antigen (EMA), cytokeratin 7 (CK7), and cytokeratin 8/18 (CK8/18) (CAM 5.2), whereas S-100 and Melan-A were negative (Figure 4). A diagnosis of EMPD was made. No evidence of metastasis was found by the multidisciplinary team, including dermatology, medical, and surgical oncology. Age-appropriate cancer screening was negative.

Wide local excision (WLE) and lymph node dissection were performed. Postoperative pathological assessment demonstrated the origin of the intraepidermal neoplastic cells from apocrine glands. Surgical margins, as well as 15 lymph nodes, were free of neoplastic cells. A diagnosis of EMPD secondary to apocrine carcinoma was made.

## Discussion

The histogenesis of primary EMPD remains controversial. Its defining cell type, the Paget cell, may arise from intraepidermal apocrine sweat ducts [8]. Mammary gland-related cells, termed Toker cells, which can be found in the nipple and vulvar epidermis, exhibit a similar immunophenotype to Paget cells, suggesting a potential shared origin [9]. Secondary EMPD represents epidermotropic spread from an underlying internal malignancy [10]. Most secondary cases originate from visceral carcinomas, particularly of urogenital or colorectal origin [3], although adnexal neoplasms have also been reported [4-7]. EMPD is an uncommon condition that predominantly affects older White and Asian adults [1,3,10-12].

The primary lesion is an erythematous plaque of variable size. Additional findings include nodules, erosion or ulceration, hypo- and/or hyperpigmentation, eczema, scaling, and crusting. Lesions are slowly progressive and commonly mistaken for more common inflammatory or infectious dermatoses [1,2,10]. Areas of red and white color have been compared to “strawberries and cream” [13]. The most common symptom is pruritus, but burning pain, tenderness, and anesthesia may also occur [11]. Ten percent of patients are asymptomatic [11], particularly those with axillary disease - up to 75% - potentially delaying diagnosis [12].

Milky-red areas were the only structures able to differentiate EMPD from Bowen disease on dermoscopy; these corresponded to histologically identified vascular proliferation and were observed in our patient [14]. Subsequently, lava lake structures and cloud-like, structureless areas were proposed. Lava lake structures consist of branching white lines and intermingling clods; cloud-like areas are made up of white, structureless areas and small, white, round clods. The latter, observed in our patient (Figure 2), corresponds to collections of tumor cells scattered along the epidermis in all directions [15].

All patients with chronically itching, eczematous, or erythematous lesions, particularly of apocrine gland-rich skin, that have been refractory to topical steroids must be biopsied [16]. The histologic hallmark, the Paget cells, are large cells with abundant, eosinophilic cytoplasm and vesicular nuclei, found singly or in clusters, and scattered throughout all layers of the epidermis [2]. Several entities can present with epidermal Paget-like cells [17]. Squamous cell carcinoma, superficial spreading melanoma in situ, and Bowen disease are the three most common intraepidermal Pagetoid neoplasms [18]. Histological criteria alone achieved statistical significance for differentiating among the three most common pagetoid neoplasms, but immunohistochemistry (IHC) might be necessary in more difficult cases [17,19]. Other authors consider IHC to be essential in distinguishing among these entities [19], and current guidelines recommend its use [1,10].

A diagnostic IHC panel for EMPD, consisting of CK7-positive, CK20-positive or CK20-negative, p63-negative, SRY-box transcription factor 10 (SOX10)-negative, and carcinoembryonic antigen (CEA)-positive results, is recommended by existing guidelines [1], but is not available at all sites. A panel consisting of S-100, CAM 5.2, and CK7 was able to differentiate between the most common pagetoid neoplasms of genital skin [20], and could be useful in settings with limited access to other IHC markers, such as our hospital. Immunohistochemical phenotype can also help distinguish between primary and secondary EMPD, and current guidelines recommend using CK20 and/or caudal type homeobox 2 (CDX2) stains for this purpose, as both are indicative of secondary EMPD [1]. Multiple IHC markers are available for immunostaining and are summarized in Table 1. Most of these are unavailable in resource-limited hospitals, underscoring the need for simplified IHC approaches to aid diagnosis and select patients amenable to surgery.

**Table 1:** Selected immunohistochemistry markers in extramammary Paget disease *In perianal Paget disease, GCDFP-15 and CDX-2, rather than CK7 and CK20, have shown better ability to distinguish primary from secondary involvement [21]. **MUC5AC, this mucin is generally negative in MPD and positive in EMPD [23,24]. However, its relationship to tumor invasion remains inconsistent in the literature. Some studies report strong expression in intraepidermal EMPD with reduced or absent staining in invasive lesions [25], whereas others describe the opposite pattern, with increased MUC5AC expression in invasive EMPD and in nodal metastases [26]. Δ CDX2 can be positive in perianal primary EMPD. Among CDX2-positive cases, an intestinal phenotype seems to be overrepresented and may mimic EMPD secondary to colorectal cancer, with no evidence of internal malignancy [31-34]; CDX2 can also be positive in secondary EMPD from colorectal origin and other primary cutaneous carcinomas, thus it should be used alongside other markers [34,35]. ¶ Although CK7 was once regarded as a relatively specific marker, it can also be positive in Toker cells, Merkel cells, Bowen disease, and actinic keratosis. In these situations, additional markers such as p63 can help clarify the diagnosis [38]. CK7, CAM 5.2, AE1/AE3, and EMA are common epithelial markers. GCDFP-15: gross cystic disease fluid protein-15; MUC5AC: mucin 5AC; TRPS1: transcriptional Repressor GATA binding GATA binding 1; CDX-2: caudal type homeobox 2; AE1/AE3: pan cytokeratin monoclonal antibodies; CEA: carcinoembryonic antigen

EMPD type	IHC markers
Primary	GCDFP-15* [21,22] and MUC5AC [23-26]**; TRPS1 was found to be sensitive and specific for primary EMPD in a small study [27].
Secondary	CK20 [22,28], CDX2* [21], and uroplakin II/III [29].
Both	CK7¶ [28], CAM 5.2, AE1/AE3, and epithelial membrane antigen [30]. Δ CDX2 (perianal primary EMPD [31-34]; secondary EMPD from colorectal origin [34,35]. CEA [36,37].

Our patient had a two-month history of an occasionally pruriginous and burning axillary plaque, which could have been asymptomatic for an unknown period of time. The incidental finding of this axillary lesion underscores the potential for delayed presentation. Our patient was diagnosed with EMPD of the axilla, which was found to be secondary to an underlying apocrine carcinoma. To date, only a handful of cases of EMPD arising from apocrine carcinoma have been documented [4-7].

Given the rarity of this occurrence, data to support an optimal management strategy are scarce. WLE and Mohs micrographic surgery (MMS) are both surgical alternatives for treatment, but the latter is considered first-line. Radiotherapy is also documented. For extensive, metastatic, and/or EMPD affecting poor surgical candidates, chemotherapy, targeted therapy, or topical modalities can be undertaken [1]. Given the lack of an MMS service in our hospital and the controversial role of sentinel lymph node biopsy, our patient was taken to WLE and lymph node dissection, but no evidence of nodal metastases was found.

To the best of our knowledge, this is the first case of axillary EMPD documented in Panama; there is no data on the prevalence of this condition in the Panamanian population, but a case report of vulvar EMPD found five cases of vulvar EMPD from 2010 to 2024 in the largest Panamanian cancer hospital, all with delays in diagnosis ranging from 4 to 10 years [39]. These findings suggest that strategies for earlier identification of EMPD are necessary in Panama.

## Conclusions

Despite its relative rarity, EMPD can present as an axillary lesion. Any chronically pruritic dermatosis on apocrine-rich skin, particularly one unresponsive to topical therapies, warrants biopsy. In our patient, histopathology revealed EMPD secondary to an underlying adnexal neoplasm, specifically an apocrine carcinoma. A limited immunohistochemical panel, including S-100, CAM5.2, EMA, and CK7, used in conjunction with histomorphology, was used to establish the diagnosis and guide definitive therapy. Additional reports are needed to further assess the sensitivity and specificity of this panel in our population. These markers may represent a cost-effective diagnostic strategy in hospitals where newer antibodies are not readily available.
